# Molecular docking and dynamics in protein serine/threonine kinase drug discovery: advances, challenges, and future perspectives

**DOI:** 10.3389/fphar.2025.1696204

**Published:** 2025-10-27

**Authors:** Gulam Mustafa Hasan, Taj Mohammad, Sobia Zaidi, Anas Shamsi, Md. Imtaiyaz Hassan

**Affiliations:** ^1^ Department of Basic Medical Science, College of Medicine, Prince Sattam Bin Abdulaziz University, Al-Kharj, Saudi Arabia; ^2^ Centre for Interdisciplinary Research in Basic Sciences, Jamia Millia Islamia, New Delhi, India; ^3^ Department of Biomedical Sciences, Heritage College of Osteopathic Medicine, Ohio University, Athens, OH, United States; ^4^ Centre of Medical and Bio-Allied Health Sciences Research, Ajman University, Ajman, United Arab Emirates

**Keywords:** molecular docking, molecular dynamics simulations, serine/threonine kinases, drug discovery, STK inhibitors

## Abstract

Protein serine/threonine kinases (STKs) regulate critical signaling pathways involved in cell growth, proliferation, metabolism, and apoptosis. Aberrant kinase activity is implicated in diverse human diseases, including cancer, neurodegeneration, and inflammatory disorders. Structure-based drug discovery, utilizing molecular docking and molecular dynamics (MD) simulations, has become a central strategy for identifying and optimizing STK inhibitors. In this review, we summarize recent advances and challenges in applying these *in silico* approaches to STK drug discovery. We discuss the principles, performance, and limitations of docking and MD approaches, as well as their integration with binding free-energy estimation methods. We emphasize recent methodological progress, including automated MD workflows, machine learning-driven interaction fingerprinting frameworks, and the growing adoption of hybrid docking-MD pipelines that enhance throughput and reproducibility. The review also highlights emerging directions such as computational design of heterobifunctional degraders (PROTACs) and allosteric modulators, which extend the scope of kinase targeting beyond ATP-competitive inhibitors. Quantitative examples of computational resource requirements and hit-validation rates from representative studies are summarized to contextualize the predictive power and practical feasibility of these approaches. Together, these developments demonstrate how the synergy of physics-based simulations, enhanced sampling, and machine learning is transforming MD from a purely descriptive technique into a scalable, quantitative component of modern kinase drug discovery.

## 1 Introduction

Protein kinases represent one of the most extensive and most biologically important enzyme families in the human genome (Koch and Bajorath, 2025). They exert their regulatory functions in various cellular processes, including proliferation, differentiation, apoptosis, metabolism, and responses to environmental stress, by catalyzing the transfer of phosphate groups from ATP to the hydroxyl groups of specific amino acid residues in substrate proteins (Mencalha et al., 2014). Of these, serine/threonine kinases (STKs) constitute the most abundant class, accounting for over 70% of the kinome (Johnson et al., 2023). STKs act as molecular switches that fine-tune signaling cascades to regulate cell fate (Jin and Pawson, 2012). STKs are functionally important with well-known families, such as the mitogen-activated protein kinases (MAPKs), which mediate the effects of growth factors and cytokines (Moens et al., 2013); cyclin-dependent kinases (CDKs), which control cell-cycle progression (Malumbres et al., 2009); Akt and the mammalian target of rapamycin (mTOR), which integrate nutrient and energy signals affecting survival and growth (Castedo et al., 2002); AMP-activated protein kinase (AMPK), which acts as a metabolic sensor for restoring energy homeostasis (Sharma et al., 2023); and glycogen synthase kinase-3β (GSK3β) or cyclin-dependent kinase 5 (Cdk5), which have central roles in neuronal physiology and in neurodegenerative diseases (Yu H. et al., 2023). This broad functional repertoire underlines why STKs are frequently dysregulated in diverse pathologies, including cancer (Maoz et al., 2019), metabolic disorders (Rawat et al., 2023), and neurodegenerative diseases (Kawahata and Fukunaga, 2023).

The clinical relevance of STKs is not restricted to human biology. Certain pathogenic bacteria also harbor eukaryotic-like STKs that contribute to stress responses, virulence, and antibiotic tolerance, as seen in *Klebsiella pneumoniae* (Hu et al., 2021; O'Boyle et al., 2025). KpnK kinase of K*. pneumoniae* promotes oxidative stress resistance and beta-lactam susceptibility, and HipA homologues mediate ciprofloxacin tolerance via autophosphorylation mechanisms similar to *E. coli* HipA (Srinivasan et al., 2014). While kinase research often focuses on human targets, recent findings suggest that STKs function as dual-function molecules, playing a central role in both human disease regulation and bacterial pathogenicity, thereby broadening their applicability from oncology and neurology to the fields of infectious disease and antimicrobial resistance (Li et al., 2022). The drug targetability of kinases has been further demonstrated by the impressive number of clinically successful kinase inhibitors (Attwood et al., 2021a). To date, the United States Food and Drug Administration (FDA) has approved over seventy small-molecule kinase inhibitors since 2001, with many now targeting STKs in addition to the more traditional tyrosine kinases (Ayala-Aguilera et al., 2022). Palbociclib and other CDK4/6 inhibitors, for example, are now standard treatments for breast cancer (Liu et al., 2018), and everolimus and temsirolimus, mTOR inhibitors, are used clinically in oncology and tuberous sclerosis complex (Palavra et al., 2017). The increasing number of kinase inhibitors that have entered the clinic with demonstrated efficacy or safety finds high translational relevance in STK research (Attwood et al., 2021a). It emphasizes the urgency for new approaches to overcome long-standing hurdles in STK drug discovery.

There have been many successes, but kinase drug discovery continues to face challenges (Cohen et al., 2021). Selectivity is the most significant challenge, as the ATP-binding site, the canonical target for the majority of inhibitors, is highly conserved across kinases, leading to off-target binding risk and dose-limiting toxicity (Ferguson and Gray, 2018). Resistance, especially in cancer, is another major limitation, with members in the kinase domain sometimes mutated such that they do not bind inhibitors as well, leading to relapse (Cohen et al., 2021). Additionally, the intrinsic conformational flexibility of kinases poses a challenge for inhibitor development because these enzymes can exist in many different and distinct states, for example, active *versus* inactive conformations or aspartate-phenylalanine-glycine (DFG)-in *versus* DFG-out states of the activation loop (Schwartz and Murray, 2011). The identification and targeting of allosteric binding sites away from the ATP pocket provide one solution, but this approach does require very high-resolution structural information (Govindaraj et al., 2022).

Although traditional kinomics, led by experimental high-throughput screening drug discovery pipelines, have yielded numerous leads, they readily incur high costs, are time-consuming, and lack the diversity of the chemical space they can access (Pollastri, 2011). Within this context, computational methods have developed into complementary and more rapid alternatives to experimental strategies (Khan et al., 2025). In particular, molecular docking and molecular dynamics (MD) simulations have become essential resources in kinase-targeted drug discovery (Naqvi et al., 2018). Docking is primarily used to predict the binding poses of small molecules to kinases (or similar structures) and their binding affinities, facilitating the virtual screening of large chemical libraries and the rational design of structure-activity relationships (Sousa et al., 2006). In contrast, MD simulations move beyond static docking models and consider the time-resolved flexibility of kinases and their complexes (Pikkemaat et al., 2002). Loop motions, activation states, solvent effects, and resistance-associated mutations that are poorly sampled in validated rigid docking models can also be explored (Shukla and Tripathi, 2020).

Docking and MD have been particularly useful in the initial stages of drug discovery against serine/threonine kinases (Roy et al., 2020; Ali et al., 2024; Khan et al., 2025). Docking can rapidly predict plausible binding modes of ligands while MD can refine those binding modes, assess their stability, and calculate the binding free-energy computed (e.g., via MM-PBSA or free-energy perturbation) (Vilar et al., 2008). Overall, this integrated workflow addresses the challenges of STKs, including difficulties in targeting essentially conserved ATP pockets, predicting the effects of resistance mutations, and characterizing potential allosteric sites that may not be readily apparent from static crystal structures (Lu et al., 2020). Such computational approaches are also valuable in the study of infectious diseases, as underexplored bacterial STKs represent promising targets for anti-virulence strategies and antibiotic-adjuvant therapies (Li et al., 2022).

In this respect, the current review exemplifies the role of molecular docking and MD simulations as a discovery tool in the search for drugs against STKs. Here, we begin with an account of the structural and functional characteristics of the STKs, before proceeding to the specifics of the docking techniques and MD simulations, and how they can be integrated into drug discovery pipelines. We then discussed the main unresolved challenges, including selective mutagenesis, conformational heterogeneity, and computational cost and scoring, followed by future perspectives on machine learning (ML)-augmented simulations, hybrid quantum mechanical methods, and experimental structural biology methods such as cryo-electron microscopy. Through integration of recent case studies with methodological advancements, this article aims to deliver a unified narrative of how computational approaches are transforming therapeutic discovery against STKs in human and microbiome-related systems.

## 2 Structural and therapeutic significance of serine/threonine kinases

STKs occupy a central role in cellular signaling because they phosphorylate serine or threonine residues on substrate proteins, thereby regulating downstream pathways that govern proliferation, differentiation, apoptosis, stress responses, and metabolism (Johnson et al., 2023). STKs contain a highly conserved bilobal catalytic domain characteristic of the kinase superfamily (Hardie, 1999). The smaller N-terminal lobe is predominantly β-sheet, containing the glycine-rich loop that stabilizes ATP-binding and the highly conserved lysine responsible for interaction with the phosphate groups of ATP (Roskoski, 2010). The C-terminal lobe, which is mainly α-helical, is substantially larger than the N-terminal lobe and forms the peptide substrate-binding interface. Within this conserved fold, multiple motifs are essential for catalysis and are also hot spots for anti-protein kinase drug design. It contains the hinge region, which binds ATP by hydrogen bonds and is a common binding position for inhibitors. Conformational changes in the activation loop switch kinases on and off (Gizzio et al., 2022). They are the primary determinants of the general state of kinase conformation and control the orientation of the magnesium ion necessary for catalysis, as seen in the DFG motif (Kung and Jura, 2016). Finally, the catalytic lysine in the β3 strand and a conserved glutamate in the αC-helix together position ATP for phosphotransfer. Such structural signatures both mediate kinase function and underpin the development of rational inhibitors.

STKs are pivotal nodes in signaling networks, and thus, they are involved in various human diseases (Capra et al., 2006). The aberrant signaling through kinases like CDKs, MAPKs, Akt, and mTOR in cancer is a major contributor to driving uncontrolled proliferation, genomic instability, angiogenesis, and evasion of apoptosis (Stefani et al., 2021). As is well-known, CDK4/6 inhibitors like palbociclib have changed the treatment landscape for hormone receptor-positive breast cancer (Liu et al., 2018). In contrast, mTOR inhibitors such as everolimus have approvals in breast cancer, renal cell carcinoma, and tuberous sclerosis (Palavra et al., 2017). The MAPK pathway kinases, particularly the ERK subfamily, remain among the most extensively studied targets in the field of oncology (Braicu et al., 2019). In tauopathies, non-receptor kinases such as GSK3β and Cdk5 play crucial roles in tau hyperphosphorylation, synaptic failure, and neuronal demise, making them attractive therapeutic targets for Alzheimer’s disease, Parkinson’s disease, and related disorders (Yu H. et al., 2023). AMPK is a cellular energy sensor that modulates ATP levels by inducing catabolic pathways, making it a well-studied therapeutic target for the treatment of obesity, type 2 diabetes, and metabolic syndrome-related diseases (Cao et al., 2025). In addition, STKs also influence inflammation, cardiovascular signalling, and immune reactions, expanding their clinical relevance (Mazzaschi et al., 2021).

The significance of STKs extends beyond human diseases and is equally intriguing in bacterial systems (O'Boyle et al., 2025). Eukaryotic-like STKs play roles in antibiotic resistance and virulence in some bacteria, including *K. pneumoniae* (Srinivasan et al., 2014). For example, KpnK modulates stress adaptation and increases β-lactam resistance, and a HipA homologue has been shown to confer a biphasic response to ciprofloxacin via autophosphorylation (Li et al., 2022). These kinases are potential new antimicrobial targets that may lead to the selective attenuation of virulence or even the potentiation of currently used and inefficient antibiotics by targeting bacterial STKs (Li et al., 2022). The action of STKs as both therapeutic in human cells and anti-virulence in pathogenic organisms positions them at the unique intersection of oncology, neurology, metabolism, and infectious disease. Although STKs are attractive drug targets, the high selectivity and potency of STK inhibitors pose a challenge in drug discovery. The primary challenge among these is the extreme conservation of their ATP-binding sites, which poses a challenge for designing molecules to selectively target closely related kinases without compromising their selectivity *versus* others (Serafim et al., 2022). As a consequence, there is often off-target toxicity due to this lack of selectivity. Another key problem is the development of resistance, especially in the field of oncology, where missense mutations in the kinase domain may decrease the affinity of inhibitors (Lu et al., 2020). These resistance mutations frequently target the gatekeeper residue, the activation loop, or the DFG motif, reshaping the kinase conformational landscape.

**FIGURE 1:**
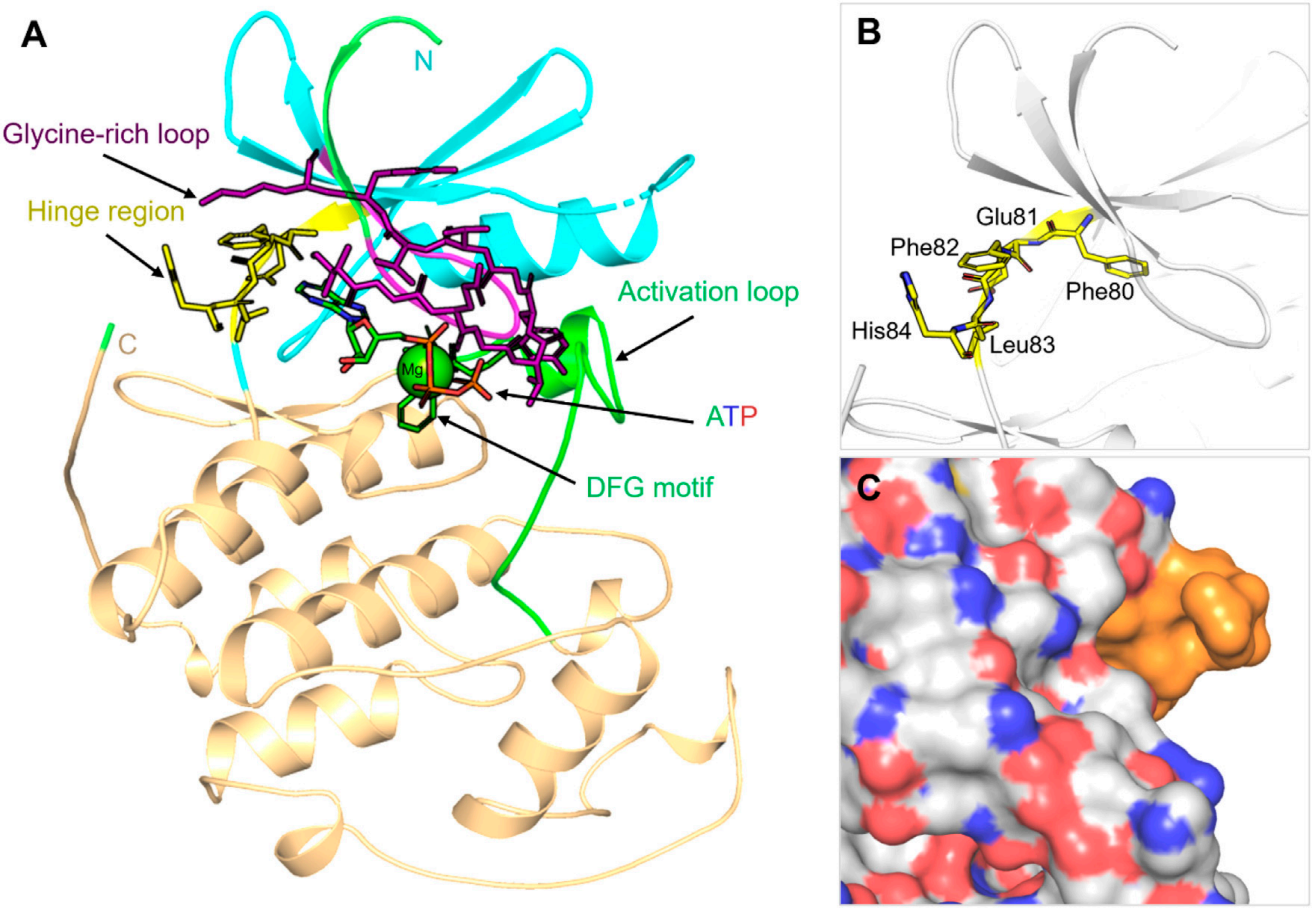
Structural architecture of serine/threonine kinases (STKs). **(A)** Representative STK catalytic domain (Cyclin-dependent kinase 2 (CDK2), PDB 1HCK) showing the conserved N-lobe (cyan), C-lobe (light orange), ATP-binding cleft and hinge (yellow), glycine-rich loop (purple), DFG motif (dark green), and activation loop (green). The bound ligand (magenta) illustrates canonical ATP-site engagement. **(B)** Close-up of hinge interactions highlighting the characteristic hydrogen-bond network that mediates broad ATP-competitive inhibitor binding. **(C)** Surface view of CDK2 reveals potential allosteric regions adjacent to the ATP pocket, highlighting the existence of cryptic binding sites that can be exploited for selectivity beyond the highly conserved ATP cleft. Structures were generated through PyMOL (DeLano, 2002) from the Protein Data Bank (Burley et al., 2022) entry 1HCK.

The intrinsic plasticity of these enzymes is another complicating factor, as kinases can exist in several different conformations following ligand binding or phospho-acceptor binding events (Hudmon and Schulman, 2002). Such conformational plasticity not only makes inhibitor design challenging but also complicates the computational prediction of efficacy, as static crystal structures typically do not represent the entire breadth of kinase states. STKs are characterized by their bilobal catalytic architecture, as well as their ATP-binding cleft and hinge region (Hardie, 1999). Conserved motifs, including the glycine-rich loop, the DFG sequence, and the activation loop, control nucleotide binding and catalysis, while the hinge provides a key hydrogen-bonding platform for inhibitor recognition. The conservation across kinases is extensive, and many ATP-competitive inhibitors target the same hinge interactions, making selective targeting challenging. However, variable and transient regions such as cryptic, allosteric pockets on kinase surfaces also provide attractive opportunities for targeting specificity. As shown in Figure 1, these architectural features are prominent in CDK2 and include numerous conserved hinge contacts and possible allosteric opportunities adjacent to the ATP-binding site (PDB: 1HCK).

Although allosteric sites provide windows for selectivity, these sites are rarely constitutive and are often difficult to identify without the use of sophisticated structural or computational techniques (Lu et al., 2014). When viewed collectively, STKs offer both significant opportunities and challenges to drug discovery. Although their centrality in disease biology validates them as excellent therapeutic targets, the structural conservation of their catalytic domains, their conformational heterogeneity, and their propensity for resistance mutations will require novel strategies. Methods that take advantage of computational approaches, including molecular docking and MD simulations, are increasingly bridging this gap by providing insights of kinase specificity, conformational flexibility, and inhibitor optimization that cannot be easily achieved through experiments (Naqvi et al., 2018). To highlight the richness and potential therapeutic relevance of STKs, Table 1 summarizes the major families of STKs, their representative members, biological functions, and provides examples of both approved and investigational inhibitors.

**TABLE 1:** Major families of protein serine/threonine kinases (STKs), representative members, their biological functions, and associated disease relevance.

STK family	Representative kinases	Biological role	Disease relevance/Therapeutic area	Remarks/Inhibitor examples
AGC family	PKA, PKB/Akt, PKC, mTOR	Cell survival, metabolism, and growth signaling	Cancer, metabolic disorders, tuberous sclerosis	Everolimus, Temsirolimus (mTOR); Perifosine (Akt)
CAMK family	CaMKII, AMPK, DAPK	Calcium signaling, energy sensing, and apoptosis regulation	Neurodegeneration, type 2 diabetes, stroke	Metformin (indirect AMPK activator); experimental DAPK inhibitors
CMGC family	CDKs, MAPKs, GSK3, CLK	Cell cycle control, stress response, neuronal regulation	Cancer, inflammation, Alzheimer’s, Parkinson’s disease	Palbociclib (CDK4/6), Trametinib (MAPK/MEK), Tideglusib (GSK3β, experimental)
STE family	MAPKKK kinases	Regulation of MAPK cascades	Cancer, immune, and inflammatory signaling	Indirectly targeted via MAPK/ERK pathway inhibitors
TKL family	MLK, MLKL	Developmental pathways, necroptosis	Inflammatory diseases, neurodegeneration	Necrostatin-1 (RIPK1 inhibitor, experimental)
RGC family	Guanylate cyclase kinases	cGMP-dependent signal transduction	Cardiovascular disease, metabolic disorders	Few selective inhibitors; potential in vascular biology
Bacterial STKs	HipA, KpnK (*K. pneumoniae*)	Stress response, virulence, and antibiotic tolerance	Antimicrobial target; drug resistance modulation	Novel target class; inhibitors under preclinical exploration

Approved and investigational inhibitors are also highlighted, emphasizing the broad therapeutic spectrum of STKs, in oncology, neurology, metabolism, and infectious disease.

## 3 Molecular docking approaches in STK inhibitor discovery

### 3.1 Principles of docking

Molecular docking is a structure-based computational method that predicts the binding mode and affinity of small molecules in the active site or allosteric site of proteins (Vilar et al., 2008). In the case of kinases and especially STKs, most docking studies have concentrated on the ATP-binding pocket, which is the most conserved and pharmaceutically targetable site in kinases (Ikram et al., 2019). Docking involves two main steps: (i) sampling, which generates possible ligand poses, and (ii) scoring, which evaluates these poses using scoring functions (Trott and Olson, 2010). In general, sampling algorithms aim to consider all possible orientations and/or conformations of a ligand with respect to the protein binding site. In contrast to rigid docking, flexible docking allows for partial rearrangements of side chains or backbone elements, simulating the features of the induced fit (Mohanty and Mohanty, 2023). However, this inherent flexibility of kinases could be dealt with better in advanced ensemble docking approaches that include multiple receptor conformations, typically obtained from crystallographic or MD simulation studies. Scoring functions estimate binding affinities to rank binding poses generated from docking calculations (Hassan et al., 2017). They can be empirical, knowledge-based, or derived from molecular mechanics force fields, and evaluate the contributions of hydrogen bonding, hydrophobic bulk interactions, electrostatics, and van der Waals packing (Trott and Olson, 2010). While scoring functions are useful, they are approximate and may not accurately recapitulate experimental binding energies. To address these limitations, consensus scoring (combining multiple scoring functions) or post-docking refinement (using MM-PBSA calculations, for instance) is widely employed (Wang et al., 2019).

### 3.2 Docking applications in STKs

Docking plays a pivotal role at multiple stages of STK inhibitor discovery (Zhong et al., 2022). Virtual screening is one of the most common applications, where thousands of chemical libraries are docked into the binding pocket of a kinase to identify useful scaffold hits (Mohammad et al., 2020b). Such a strategy minimizes the number of candidates to be validated experimentally, saving time and cost (Alrouji et al., 2025). Docking also supports binding mode prediction, enabling the visualization of inhibitor binding to important kinase motifs, including the hinge region, the conserved catalytic lysine, or the DFG motif. Such information is useful for understanding structure-activity relationships and aiding the next steps in medicinal chemistry. A further obvious application is selectivity profiling, where candidate inhibitors are docked against panels of related kinases (Zhong and Almahmoud, 2023). Selectivity is a major challenge in kinase drug design, as the ATP-binding site is highly conserved across the kinome, making docking-based profiling a valuable first step towards predicting off-target interactions that can be subsequently tested experimentally. In the case of specifically in STKs, docking has been used to develop inhibitors that capitalize on minor variations in shape and electrostatics of the binding pocket.

Docking analyses have guided the identification of compounds that specifically bind CDK4/6 relative to other CDK isoforms and have also revealed key interactions in the hydrophobic pocket next to the hinge region in mTOR inhibitors (Najmi, 2025). Docking is also increasingly applied to drug repurposing, where existing FDA-approved drugs are screened against STK targets to find new possible therapeutic uses. This strategy is particularly attractive, as the pharmacokinetic and safety profiles of repurposed drugs have already been determined, allowing for a more rapid translation to the clinic.

### 3.3 Docking success stories in kinase drug discovery

Several landmark examples highlight the importance of docking in drug discovery for kinases (Attwood et al., 2021a). Imatinib, such a targeted agent, is actually a pan-tyrosine kinase inhibitor and serves as a paradigm for future STK inhibitor development (Di Vito et al., 2023). BCR-ABL is in the autophosphorylated state, and docking studies have shown that imatinib stabilizes the inactive conformation by forming hydrogen bonds with the hinge region and binding in the hydrophobic back pocket exposed in the DFG-out state (Rocha et al., 2021). The success of this was translated to STKs, where inhibitors were similarly optimized to exploit conformational states. For example, docking-guided structure-activity relationship studies were instrumental in identifying and optimizing hinge-binding motifs that imparted isoform selectivity in the case of CDK inhibitors, such as palbociclib and ribociclib (Braal et al., 2021). In recent years, however, docking-based drug repurposing has found surprising interactions of approved drugs on STKs (Wang et al., 2024).

Recently, antidiabetic drugs that activate AMPK have been repurposed, and several anticancer agents have been experimentally validated as mTOR inhibitors (Khan et al., 2024). Such success stories highlight both the power (to generate structural hypotheses) and the weaknesses of docking. Docking predictions were often refined with MD simulations and/or validated using crystallography and biochemical assays (Huang and Hu, 2025). However, docking remains the initial step in the computational pipeline for discovering kinase inhibitors, providing a rapid screen of vast chemical spaces, insight into binding interactions, and aiding in the rational design of more potent and selective inhibitors. Several software platforms are available for performing docking studies of kinases, each with its own merits and demerits, and therefore preferred for specific applications. An overview of the docking programs frequently used for various types of proteins, including STKs, is given in Table 2.

**TABLE 2:** Widely used molecular docking software platforms for kinase inhibitor discovery, including open-source and commercial tools.

Software	Type	Sampling method	Scoring function	Strengths	Limitations	Applications in kinase studies
InstaDock	Open-access (GUI for QuickVina-W)	Flexible ligand, semi-rigid receptor	Vina scoring	User-friendly GUI; batch screening; accessible to non-programmers	Limited receptor flexibility; less customizable	Virtual screening of large libraries; kinase-focused repurposing screens
AutoDock/AutoDock Vina	Open-source	Lamarckian genetic algorithm (AutoDock); gradient optimization (Vina)	Empirical free energy scoring	Widely used; flexible ligand; semi-rigid receptor; good community support	Scoring function relatively simple; limited allosteric handling	Broad kinase inhibitor screening; hinge-binding motif analysis
DOCK	Open-source	Grid-based matching	Force-field based	Early and efficient tool; handles large libraries well	Older interface; less advanced handling of protein flexibility	Used in early MAPK and CDK docking campaigns
Glide (Schrödinger)	Commercial	Systematic search with grid-based potentials	GlideScore	High accuracy; multiple precision modes (HTVS, SP, XP)	Proprietary; requires license; high cost	Benchmark kinase inhibitor design; hinge region SAR optimization
GOLD	Commercial	Genetic algorithm	ChemScore, ASP, GoldScore	Robust handling of ligand flexibility; reliable for kinases	Proprietary; performance depends on the scoring function	Selectivity profiling across kinase families (e.g., CDKs, MAPKs)
CDOCKER (Discovery Studio)	Commercial	CHARMm-based MD docking	Force-field based	Explicit receptor flexibility; MD refinement of docking	Limited to the Discovery Studio platform; license required	Applied to mTOR and CDK inhibitor optimization
RosettaLigand	Open-source	Monte Carlo + minimization	Rosetta energy function	Good induced-fit handling; flexible docking	Complex workflow; steeper learning curve	Allosteric site exploration in STKs; flexible loop docking

Each entry summarizes the sampling method, scoring function, strengths, and limitations, with representative applications in serine/threonine kinase (STK) research.

### 3.4 Choosing docking strategies for orthosteric vs. allosteric/cryptic pockets

For STKs, the ATP (orthosteric) site is well-defined and generally well-handled by grid-based and standard flexible ligand docking approaches that assume limited receptor rearrangement. Tools such as AutoDock Vina and Glide (HTVS/SP) are efficient for large-scale orthosteric virtual screening and hinge-motif SAR exploration (Trott and Olson, 2010). In contrast, allosteric and cryptic pockets typically require explicit receptor flexibility or ensemble approaches. Methods such as induced-fit methods (e.g., RosettaLigand, GOLD with flexible sidechains, Glide Induced-Fit), MD-derived ensemble docking, or MD-refined docking (e.g., CDOCKER with MD refinement) are more suitable (DeLuca et al., 2015; Wu and Brooks III, 2021). For cryptic pockets that open transiently, generating receptor conformations by enhanced sampling MD (metadynamics, GaMD, replica-exchange) or by short, targeted MD, then using ensemble docking across those conformations is recommended (Kuzmanic et al., 2020b). Finally, consensus and rescoring strategies, e.g., docking, short MD, MM-GBSA rescoring, often perform best when seeking selective allosteric modulators.

## 4 Molecular dynamics simulations in STK inhibitor design

### 4.1 Fundamental role of MD in kinase studies

Molecular docking provides a quick perception of potential ligand binding orientations within protein active sites. However, docking assumes a relatively static protein structure and fails to capture the full dynamic range of kinases. STKs are highly flexible enzymes, like other members of their superfamily; the transition between different conformations is an essential part of their function (O'Boyle et al., 2025). This includes changes in the conformation of the activation loop, the glycine-rich P-loop, and the DFG motif, which can result in rapid and sometimes large alterations of ligand accessibility to the binding pocket and/or the binding affinity of the ligand to the target (Schwartz and Murray, 2011). MD simulations overcome these limitations by solving Newton’s equations of motion for systems of atoms and by offering time-resolved, atomic-time trajectories of protein-ligand complexes (Fu et al., 2022). MD simulations allow exploration of broader aspects of protein flexibility, solvation, ion coordination, and inter-residue water-mediated interactions that are seldom present during docking studies. MD allows the user to observe how a kinase toggles between these states, how an inhibitor stabilizes or destabilizes those states, and whether water molecules play a role in essential hydrogen bonding networks in the binding site. Crucially, MD tests docking-derived poses for stability under physiological conditions, which ensures that such interpretations of binding modes are not merely artifacts of rigid docking algorithms.

### 4.2 Key applications

There has been a growing application of MD to STK drug discovery in recent years, and multiple different roles have emerged (Attwood et al., 2021b). One important application is docking pose validation (Alzain et al., 2025). MD simulations in an explicit solvent can also be used to relax the protein-ligand complex and explore whether the interactions remain stable over nanosecond to microsecond time scales after a successful docking experiment, suggesting potential inhibitors (Roy et al., 2020). Stable trajectories imply real predictions of docking, rapid dissociation of the ligand, or significant rearrangements of the complex indicate a false positive. MD is also fundamental to the crystallographic analysis of the conformational flexibility of STKs (Gizzio et al., 2022). Kinases frequently toggle between DFG-in and DFG-out configurations, as well as open and closed states of the activation loop or inward- and outward-facing conformations of the αC-helix. They help determine whether inhibitors can bind to active or inactive conformations, and as such are crucial to the design of inhibitors. Simulations illustrated mechanisms at an atomic level, explaining how the inhibitors bias protein kinases to use the inactive conformation over the active conformation.

A third key application is the investigation of resistance mutations (Yu Y. et al., 2023). Mutations that change the conformational dynamics or steric environment of the binding pocket often leads to clinical resistance. MD simulations have been utilized to model these mutations, indicating changes in hydrogen bonding networks, disruptions in hydrophobic packing, and alterations in inhibitor-bound state stability (Mohammad et al., 2020a). While the extensive literature on resistance mechanisms has targeted tyrosine kinases, such as EGFR or BCR-ABL, the same paradigms are relevant to STKs, as resistance mutations can limit the clinical utility of CDK or mTOR inhibitors (Alves et al., 2021). MD may also be one of the most valuable tools for the discovery of allostery (Govindaraj et al., 2022). In contrast to ATP-competitive inhibitors that target the conserved catalytic pocket, allosteric inhibitors utilize non-catalytic, often transient sites. Such sites are hard to discern with static crystallography but are well exposed by long MD simulations that can reveal opening and closing motions or expose cryptic pockets. Simulations of mTOR have, for instance, revealed hydrophobic pockets that lie outside the canonical ATP-binding site, which are currently being explored for their potential as allosteric regulators (Nunes Azevedo et al., 2023).

### 4.3 Recent advances

Recent methodological and computational advances have greatly improved the utility of MD for targeted multi-scale drug discovery against kinases (Sadybekov and Katritch, 2023). Meanwhile, GPU acceleration, or the availability of specialized hardware (such as Anton supercomputers), allowed the extension of the simulation time window from nanoseconds to microseconds and even milliseconds (Shaw et al., 2021). Such extended simulations enhance conformational sampling and capture rare yet biologically relevant transitions, such as activation loop unfolding or ligand unbinding events. Moreover, this qualitative understanding of ligand binding has been coupled with several binding free-energy methods on MD, and this has allowed for an increasingly quantitative prediction of inhibitor affinity. Molecular Mechanics/Poisson-Boltzmann Surface Area (MM-PBSA) and Molecular Mechanics/Generalized Born Surface Area (MM-GBSA) are post-processing approaches that facilitate fast, albeit approximate, binding free energy calculations from MD trajectories (Wang et al., 2019). Therefore, more rigorous alchemical methods, such as free energy perturbation (FEP) and thermodynamic integration, yield higher accuracy but also at a significantly larger computational expense (Ruiz-Blanco and Sanchez-Garcia, 2020).

In practice, MM-PBSA and MM-GBSA are applied as end-point estimators on snapshots extracted from production MD trajectories (Genheden and Ryde, 2015). Typical workflows perform energy decomposition to parse contributions from van der Waals, electrostatic, polar solvation (using PB or GB), and nonpolar solvation. These methods are computationally inexpensive relative to alchemical FEP/TI and are therefore widely used to re-rank docking hits and to prioritize analogues for synthesis. However, MM-PBSA/MM-GBSA accuracy depends strongly on sampling quality, choice of dielectric and surface tension parameters, and force-field consistency between MD and ligand parameterization. For kinases, where solvent networks and flexible loops can substantially influence binding energetics, it is advisable to extract energies from multiple independent replicate simulations to quantify statistical uncertainty, to report the mean and standard deviation of the calculated Δ*G* values, and to validate MM-PBSA/MM-GBSA results against at least a subset of experimental affinities before relying on them for decision making. When higher accuracy is required during lead optimization, alchemical free-energy methods such as FEP or TI remain the benchmark approaches despite their greater computational cost.

Other notable advances are enhanced sampling techniques (Lazim et al., 2020). Kinetic traps frequently constrain standard MD, as proteins can reside entrapped in local conformations that may not reflect the complete conformational landscape (Kuzmanic et al., 2020a). Now, we have methods such as accelerated MD, metadynamics, replica-exchange MD, and Gaussian accelerated MD to bypass these barriers, unveiling hidden conformations and improving conformational sampling (Wang et al., 2021). The advantages of these methods have previously helped dissect activation loop dynamics, pinpoint cryptic allosteric sites, and study conformational selection during ligand binding in kinases (Kuzmanic et al., 2020a). Lastly, the trends of MD with structural biology and artificial intelligence (AI) are future directions in kinase studies (Agajanian et al., 2023).

Importantly, the last few years have seen tangible improvements in throughput, automation, and downstream analysis of MD-based hit refinement. Automated MD pipelines that streamline setup, execution, and post-processing of many protein-ligand simulations now exist and have been applied to accelerate hit prioritization (Brueckner et al., 2024). Examples include Admiral, an automated docking, MD, and analysis platform that orchestrates simulation setup, runs, and automated reporting for medicinal chemistry teams, and recent automated MD workflows that integrate ML models to generate per-ligand simulation fingerprints and prioritize candidates (Baumgartner and Zhang, 2020). Complementary to automation, tools for encoding molecular interactions from MD trajectories as compact fingerprints have facilitated rapid comparisons and ML-driven analyses. Libraries such as ProLIF enable the extraction of interaction fingerprints from trajectories and trajectory-derived ensembles, allowing clustering of ligand binding modes, feature engineering for ML models, and rapid filtering of MD-derived poses (Bouysset and Fiorucci, 2021).

When combined with automated MD workflows and adaptive sampling, interaction fingerprinting supports scalable, reproducible post-processing of large MD datasets and enhances the interpretability of ML models trained on dynamic interaction patterns. MD simulations have become a staple in providing dynamic context to structures obtained from experimental methods such as cryo-electron microscopy, NMR, and X-ray crystallography (Son et al., 2024). This led to the employment of ML approaches that utilize large MD datasets to pull out essential collective variables and expedite the conformational sampling process (Wang et al., 2020). This, in turn, enhances the reach and precision of MD, making it a cornerstone in rational STK inhibitor discovery. There are various MD packages, each with specific pros and cons that limit their application to kinase simulations. Conventional MD engines and their applications in the discovery of STK inhibitors are summarized in Table 3.

**TABLE 3:** Major molecular dynamics (MD) software packages employed in kinase simulations.

Software	License	Strengths	Limitations	Applications in kinase research
GROMACS	Open-source	Swift; strong GPU acceleration; widely used in academic labs; large community support	Limited force-field variety compared to AMBER/CHARMM; less intuitive for absolute binding free-energy methods	Validation of docking poses; long-timescale simulations of MAPKs, CDKs, and mTOR; widely used for MM-PBSA in kinase-ligand studies
AMBER	Commercial/academic licenses	Rich library of biomolecular force fields (ff14SB, GAFF); strong MM-PBSA/MM-GBSA support; good integration with quantum mechanics (QM/MM) tools	Slower than GROMACS for extensive systems; license restrictions for some components	Free-energy calculations for CDKs and Akt inhibitors; QM/MM studies of catalytic residues in STKs
NAMD	Open-source	Highly scalable on large clusters; efficient CHARMM force field support; good for extensive systems	Moderate learning curve; less user-friendly for beginners	Long-timescale simulations of kinase conformational changes (e.g., DFG-in/out transitions); ensemble simulations for inhibitor selectivity
CHARMM	Commercial (academic version available)	Highly detailed biomolecular modeling; extensive force-field options; strong for advanced free-energy methods	Complex input and steep learning curve; less streamlined than GROMACS/AMBER	Detailed mechanistic studies of ATP binding in kinases; conformational plasticity analysis of STKs
Desmond (Schrödinger)	Commercial	Extremely fast; optimized for GPUs; seamless integration with Glide docking results	Proprietary; limited customizability compared to open-source tools	Kinase inhibitor optimization pipelines (Glide docking, Desmond MD refinement); mTOR and CDK inhibitor refinement
OpenMM	Open-source	Highly flexible and customizable; strong GPU acceleration; Python-based API makes integration with ML easy	Still in development; has a smaller user base; fewer validated workflows than GROMACS/AMBER.	AI-driven kinase simulations; adaptive sampling of STK conformations; emerging tool for integration with ML-enhanced workflows

The table outlines license type, strengths, limitations, and representative applications in studying serine/threonine kinase (STK) structure, conformational flexibility, and inhibitor binding.

## 5 Integrative docking-MD workflows in STK drug discovery

### 5.1 Docking as the first step

Rational drug discovery can greatly benefit from a stepwise integration of molecular docking and MD simulations, which are complementary approaches today (Sadybekov and Katritch, 2023). Docking is typically employed as an initial step due to its speed and generality in screening large compound libraries against kinase targets. The ability to effectively explore vast chemical spaces and identify possible ligand binding poses, ranking them based on scoring functions, allows docking to help researchers effectively narrow down chemical spaces to a reasonable subset of candidates (Huang and Hu, 2025). This is especially useful for STKs for which the experimental high-throughput screening is expensive and inefficient due to the similarity of the conserved ATP-binding site (Zhang et al., 2022). Docking has the potential to identify ligands that utilize small differences in hinge regions, hydrophobic pockets, or allosteric cavities, and to create testable hypotheses about selectivity and potency, guiding downstream computational and experimental assays.

**FIGURE 2:**
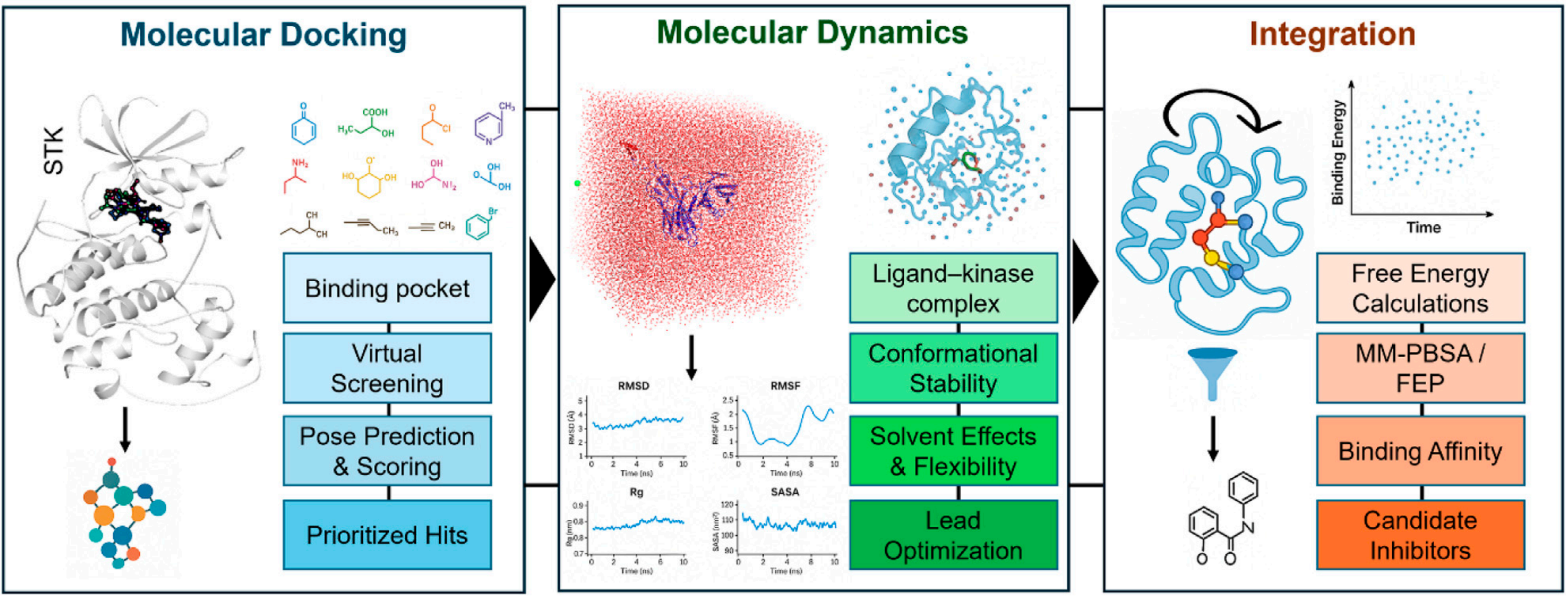
Schematic overview of an integrated computational pipeline for serine/threonine kinase (STK) inhibitor discovery. Molecular docking (left) enables the identification of the binding pocket, virtual screening of chemical libraries, prediction of binding poses, scoring, and prioritization of hits. Molecular dynamics simulations (middle) refine docking predictions by evaluating ligand-kinase complex stability, conformational flexibility, solvent effects, and lead optimization. Integration of docking and MD (right) allows free energy calculations (e.g., MM-PBSA, FEP), estimation of binding affinities, and selection of stable candidate inhibitors for experimental validation. Together, these complementary approaches provide both breadth (docking-based exploration) and depth (MD-based refinement) in kinase-targeted drug discovery.

Molecular docking and MD simulations constitute complementary approaches that, when combined, offer a high-throughput and evidence-driven pipeline for kinase-targeted drug discovery. Docking acts as a quick initial layer for virtual screening and pose prediction. In contrast, MD then refines these predictions in physiologically relevant environments, permitting the inclusion of protein flexibility, solvent effects, and dynamic stability. Together, they enable better predictive power for binding depth and more effective prioritization of candidate inhibitors. Figure 2 provides a schematic overview of this integrated workflow in the context of STK inhibitor discovery.

### 5.2 MD for refinement and validation

MD simulations are used to identify, characterize, and validate promising compounds through docking under dynamic and physiologically relevant conditions after all compounds have been docked (Lazim et al., 2020). While docking usually considers the protein rigid, MD considers the conformational flexibility of both ligand and receptor, and also the solvent effects and long-range electrostatics. In the final stage, the stability of docking poses is evaluated using molecular dynamics simulations of the protein-ligand complex in explicit solvent, spanning nanosecond to microsecond timescales (Fu et al., 2022). If the docking-predicted pose remains stable, the inhibitor is more likely to be a true binder; conversely, ligand dissociation or major conformational rearrangements may indicate a false positive. MD further enables side chains in flexible kinase motifs, such as the activation loop, P-loop, or DFG motif, to relax and fit the binding ligand, providing more realistic perspectives on binding (Shukla and Tripathi, 2020). The other improvement step is to extract the binding free energies from MD trajectories. In addition to re-ranking docking hits with approximate approaches such as MM-PBSA or MM-GBSA, alchemical methods like FEP permit quantitative affinity predictions (Wang et al., 2019; Ruiz-Blanco and Sanchez-Garcia, 2020). This is useful in avoiding some of the biases of docking scoring functions, which are generally poorly or only moderately related to experimental binding affinities. The combined use of docked hits and MD-based free energy calculations enables the generation of a reliable ranking of potential inhibitors, which can effectively limit the number of compounds to be synthesized and tested experimentally.

### 5.3 Example workflows and case studies

The effects of this docking and MD harmony are best seen with combined workflows (Shaikh et al., 2023). The standard pipeline starts with docking large libraries of compounds against an STK target in virtual screening (Al-Fahad et al., 2025). This step reveals stable ligands that then undergo MD simulations to confirm their relative stability in the binding pocket. These simulations yield binding free energies that are used for ranking, and then the most promising candidates are chosen for experimental testing (Zhang et al., 2024). Such a two-pronged strategy has proven successful in discovering inhibitors for multiple STKs (Tarazi et al., 2016; Hassan et al., 2023). One recent study focusing on CDK1 initially used docking to screen commercially available databases of candidate inhibitors that were refined in ranking through MD simulations and MM-PBSA calculations (Teotia et al., 2024). Among the highest-ranked compounds, several were found to have micromolar inhibitory activity *in vitro*, thereby validating the predictions made from computational analysis. Docking was used to identify compounds that not only bind to ATP-competitive sites but also to allosteric sites in mTOR inhibitors; similar strategies have been applied (Dahiya et al., 2019; Gupta et al., 2019; Botelho et al., 2022). While MD simulations also confirmed the binding stability of these inhibitors, they revealed dynamic movements of the kinase domain that were not apparent from static docking results.

An additional illustrative example is the case of salt-inducible kinases (SIKs), where ensemble docking using MD-derived conformations improved the correlation between the predicted docking score and the log of experimental IC_50_ values (Valdés-Albuernes et al., 2025). These case studies exemplify how docking brings breadth-rapid exploration of chemical space, while MD contributes depth, dynamic validation, and energetic optimization. This represents a rational, iterative framework for kinase inhibitor discovery encompassing both docking and MD. Docking creates the first hypotheses regarding binding poses and possible selectivity. At the same time, MD interrogates and refines these hypotheses, providing insight into conformational dynamics, resistance mutations, and solvent-exclusion-mediated interactions (Tesch et al., 2021). These integrated approaches significantly enhance the efficiency and fidelity of computational pipelines, enabling translation of *in silico* predictions to validated kinase inhibitors in the lab. Due to the importance of the STKs in cellular signalling, several recent studies combining docking with MD simulations have been performed that identified and optimized their inhibitors. Hybrid workflows, summarized in Table 4, provide mechanistic insight and direct experimental validation through representative case studies.

**TABLE 4:** Representative case studies (2020–2025) of serine/threonine kinase (STK) inhibitor discovery using integrated docking and molecular dynamics (MD) approaches.

Target kinase	Computational approach	#Compounds screened (library/source)	Experimentally validated hits/Hit rate (%)	Key outcome/Findings	Therapeutic context	Year	References
CDK1	Docking + MD + MM-PBSA	288,671 (DrugBank, Selleck, Otava, in-house)	Tested: 10 Validated: 3 Hit rate: ∼30%	Identified 3 novel inhibitors with IC_50_ < 5 μM; stable hinge-binding interactions validated by MD	Cancer therapy (cell cycle inhibition)	2024	Teotia et al. (2024)
JAK1/JAK3	Pharmacophore modeling + Docking + MD	28 (Custom library) PubChem/ZINC (extensive database)	Tested: 0* Validated: 0* (2 predicted) Hit rate: N/A	Repurposed Baricitinib and Ruxolitinib scaffolds; stable dynamics confirmed	Autoimmune diseases/inflammatory disorders	2024	Faris et al. (2024)
FAK (Focal Adhesion Kinase)	Docking + MD modeling	47 designed analogs of VS-4718	Tested: 0* Validated: 0* Hit rate: 0%	Designed 47 new analogs of VS-4718; predicted improved binding affinity	Oncology (metastasis inhibition)	2022	Shi et al. (2022)
SIKs (Salt-inducible kinases 1/2/3)	Ensemble docking across MD-derived conformations	44 literature SIK inhibitors	Tested: 0* Validated: 0* Hit rate: N/A	Improved correlation (R^2^ ∼0.8) between docking scores and experimental activity	Metabolic and inflammatory disorders	2025	Valdés-Albuernes et al. (2025)
Aurora Kinase A	Docking + long-timescale MD + MM-GBSA	Pepper TRPV1 ligands (PubChem AID 624919)	Tested: 3 (*in silico*) Validated: 2 (stable in MD) Hit rate: 67%	Predicted selective natural product inhibitors; MD revealed conformational stabilization of the inactive state	Cancer therapy (mitosis regulation)	2023	Singh et al. (2023)
GSK3β	Docking + MD + free energy calculations	Virtual tetrazole library (size N/A)	Tested: 0* Validated: 0* Hit rate: N/A	Discovered dual-target neuroprotective compounds; MD confirmed stability and binding mode	Neurodegenerative disorders (Alzheimer’s, Parkinson’s)	2025	Joshi and Alavala (2025)

Each example highlights the computational strategy, main findings, therapeutic context, and year of publication. “*” indicates that the study did not report any experimental assay.

## 6 Challenges and limitations in STK computational drug discovery

Molecular docking and MD simulations have evolved the way kinase inhibitors are discovered by providing atomistic resolution in elucidating binding interactions, conformation dynamics, and resistance mechanisms (Attwood et al., 2021b). Nevertheless, despite their increasing effectiveness, some limitations still bound these models to their fullest potential, specifically regarding STKs. These simulations stem partly from fundamental properties of kinases themselves (e.g., structural conservation and conformational plasticity) and partly from the current computational and methodological limitations of *in silico* approaches. Knowledge of these limitations will be crucial in developing new solutions that enhance the predictive, cost-efficient, and translationally relevant aspects of computational pipelines. The key challenges for docking- and MD-based discovery of STK inhibitors are further discussed below.

### 6.1 Selectivity issues

One of the most durable challenges in designing STK inhibitors has been selectivity. The ATP-binding site, which represents the most common target among inhibitors, is highly conserved across the kinome (Li et al., 2021). While subtle differences can be leveraged in the hinge region, hydrophobic pockets, or neighboring residues, inhibition is often multi-targeted, resulting in off-target effects and toxicity, given the high degree of conservation. Although molecular docking can amplify these differences to an extent, it often overestimates selectivity owing to the static nature of the model. Docking scores for related kinases are often so similar that distinguishing selective from promiscuous inhibitors becomes difficult. This situation is mitigated by MD simulations, which elucidate the subtle conformational dynamics that may impart preferential binding to one kinase over another; however, the kinome-overlapping specificity remains a major challenge that is still unsolved. However, the discovery of allosteric inhibitors that bind to cryptic or transient sites outside the ATP pocket is a promising approach; however, identifying these requires long simulations and complex analysis.

### 6.2 Computation cost

Another significant bottleneck is the computational cost associated with high-quality simulations. Docking is computationally inexpensive and suited for high-throughput screening, whereas MD simulations are resource-intensive. While nanosecond-scale simulations have become standard, motions relevant to biology, such as rearrangements of activation loops, DFG flips, or the unbinding of ligands, often have entropically unfavorable long timescales on the order of microseconds to milliseconds. These events mostly require either very long simulations or enhanced sampling methods (e.g., metadynamics or accelerated MD), both of which lead to a massive increase in computational cost. GPU acceleration and specialized hardware (e.g., Anton supercomputers) have made this idea somewhat feasible, but these hardware resources are still rare (Shaw et al., 2021). The same computational barrier prevents MD from utilizing advanced methodologies in many academic and resource-limited settings, highlighting the disparity between theoretical potential and practical applicability.

Quantitatively, these costs vary with system size and hardware, although large-scale docking remains relatively inexpensive. High-throughput screening of around 250,000–300,000 compounds typically requires only a few days on a modest CPU cluster. In contrast, MD refinement becomes the major computational expense, where a 100 ns production run for one protein-ligand complex generally consumes about 20–30 GPU hours on modern hardware, so studies evaluating multiple candidates or replicates easily reach hundreds of GPU hours. Ensemble docking, where multiple receptor conformations are used, increases this cost proportionally with the number of conformers. At the same time, MM-PBSA or MM-GBSA rescoring adds only minutes to hours per complex. More rigorous FEP/TI calculations are even costlier, often requiring tens to hundreds of GPU hours per ligand pair. Consequently, researchers are encouraged to report not only the methods used but also hardware specifications, wall-clock times, and normalized compute metrics (e.g., GPU-hours or CPU-core-hours) to improve transparency and allow realistic comparisons across studies.

### 6.3 Scoring inaccuracies

Scoring functions are another crucial open issue (Barradas-Bautista et al., 2023). Experimental binding affinities are often poorly correlated with fast, but inaccurate, mathematical approximations of docking. Consequently, docking pipelines frequently yield false positives (predicted binders that are inactive in a biochemical assay) and false negatives (true binders that go undetected). The presence of highly similar hinge-binding motifs among STKs makes it difficult for scoring functions to resolve small variations in binding energetics. More accurate predictions (compared to standard docking approaches) can be obtained after refinement using MD-based binding free-energy calculations, such as MM-PBSA or alchemical FEP; however, this added accuracy comes at a cost of a several orders of magnitude increase in computational resources. Despite this refining, the predictions still rely heavily on the quality of the underlying force fields, which is especially true for metal cofactors or systems with complex solvent interactions.

### 6.4 Resistance mutations

Resistance mutations pose a significant hurdle to the development of kinase inhibitors overall and present a greater challenge in oncology (Lu et al., 2020). While primarily documented for tyrosine kinases, resistance mutations have also been described for STKs that affect these drug-binding pockets from which inhibitors cannot escape. For example, mutations in the gatekeeper residue, the activation loop, or the DFG motif can dramatically decrease the binding affinity of the inhibitor due to steric hindrance to access or destabilization of the inactive conformations targeted by some drugs (Huang et al., 2015). Unless mutant models are created, docking methods generally overlook these effects, and even then, they do not account for the dynamic consequences of mutations. MD simulations provide realistic insights into how mutations alter conformational flexibility and drug-binding landscapes; however, the high computational cost of simulating multiple mutant variants often becomes prohibitive. Moreover, because resistance evolution in the clinic is highly unpredictable, it has limited the development of enduring therapies based on computational predictions.

### 6.5 Water molecules and allostery

**FIGURE 3:**
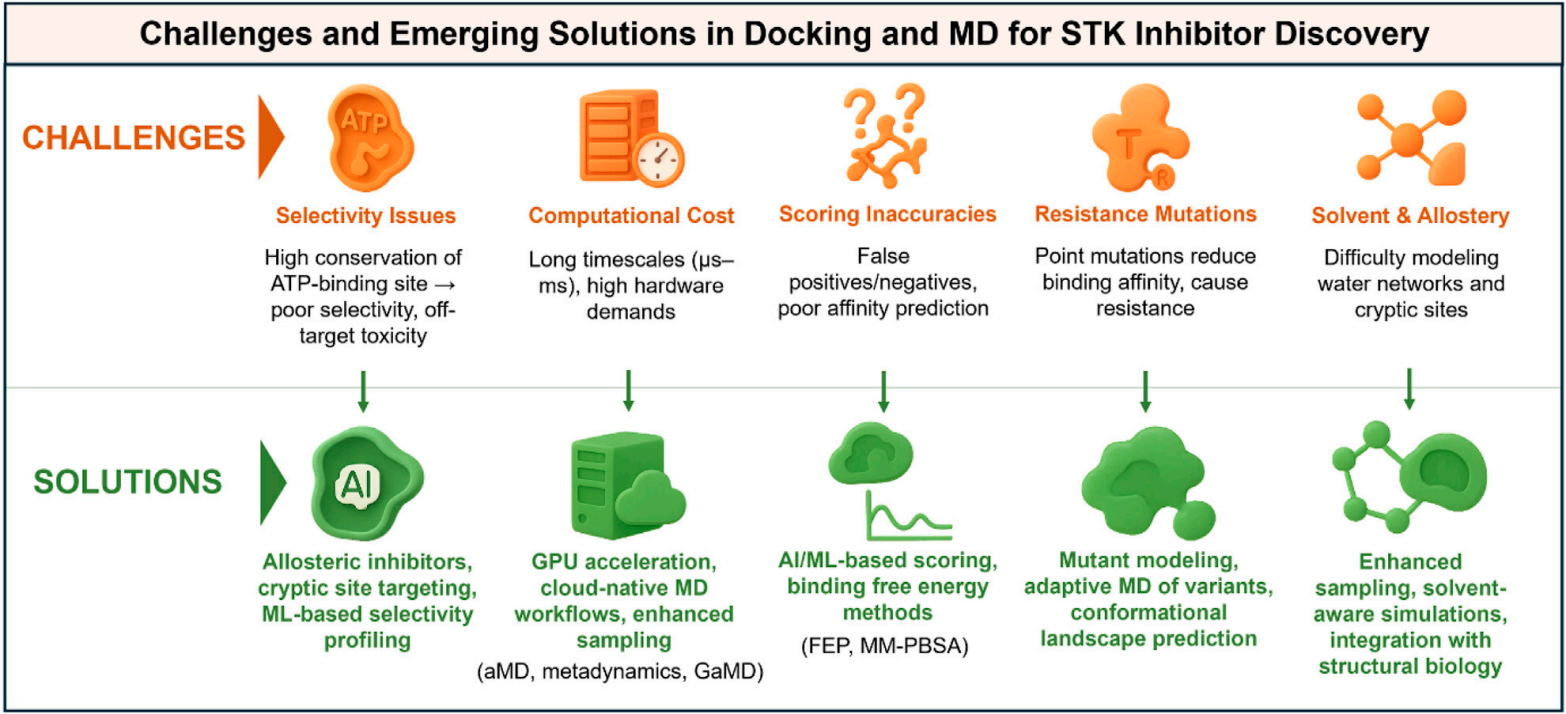
Challenges and solutions in applying docking and molecular dynamics (MD) to serine/threonine kinase (STK) inhibitor discovery. Infographic summary highlighting common challenges (left, red) and emerging solutions (right, green). Key limitations include selectivity issues arising from conserved ATP-binding pockets, high computational cost, inaccuracies in docking scoring functions, resistance mutations, and difficulties in modeling solvent and allosteric effects. Corresponding solutions include the development of allosteric inhibitors and machine learning-based selectivity profiling, as well as GPU-accelerated and cloud-native workflows with enhanced sampling methods. These solutions also incorporate AI-driven scoring and free-energy calculations, mutant modeling using adaptive MD simulations, and the integration of enhanced sampling with structural biology approaches. Together, these innovations are helping to overcome current bottlenecks and improve the predictive power of computational kinase drug discovery pipelines.

The correct treatment of water molecules and allosteric effects is still an unsolved problem. Kinase-ligand interaction is often mediated with the help of water molecules, which facilitate hydrogen bonds between inhibitors and protein residues (Zhu and Hu, 2022). However, explicit waters are either overlooked by most docking algorithms or treated very simplistically, leading to the omission of important stabilizing interactions (Samways et al., 2021). Explicit water docking remains computationally intensive and algorithmically cumbersome. However, MD simulations, which inherently account for one solvent, can provide general insight into the functionality of water network-mediated interactions; however, an unambiguous role of dynamic water networks would still be difficult to interpret. Allosteric regulation is another such wrinkle, in the same general class. Many kinases are predicted to contain cryptic or transient pockets outside the ATP site that can be used for allosteric inhibition. These types of sites are often not visible in crystal structures and are difficult to detect by docking studies. Although sites like these can be seen in long-timescale MD or enhanced sampling, predicting their druggability and designing inhibitors that exploit them has been an enormous challenge to the field. The orthosteric and allosteric sites do not act in isolation, as the dynamic interplay complicates all predicted scenarios, highlighting the necessity of an integrative approach that combines computational and experimental structural biology. Figure 3 provides a short overview of the main challenges in applying docking and MD to drug discovery against STK, and these include computational remedies to the challenges.

## 7 Summary of challenges

All these limitations together shed light on the double-edged sword of computational kinase drug discovery. Docking and MD, on the one hand, let us gain unprecedented information on inhibitor binding, conformational dynamics, and the mechanism of resistance. On the other hand, issues such as selectivity, computational expense, scoring accuracy, mutation unpredictability, and solvent or allosteric complexity still hinder their development. Further advances will come from ML-enhanced scoring and force fields, increased availability of high-performance computing, and tighter coupling between simulation and experiment. If these barriers are addressed, computational approaches will provide a more consistent input into the next-generation discovery pipeline of STK inhibitors. Docking and MD are complementary strengths that also overcome some limitations of each other. MD complements docking along a continuum of breadth *versus* depth: docking provides breadth via high-throughput virtual screening; MD provides depth via refinement of predictions in physiologically realistic conditions. Table 5 summarizes the comparative advantages, limitations, and best use cases of both approaches in the discovery of STK inhibitors.

**TABLE 5:** Comparative advantages, limitations, and best use cases of molecular docking *versus* molecular dynamics (MD) simulations in serine/threonine kinase (STK) drug discovery.

Aspect	Molecular docking	Molecular dynamics (MD)
Speed and scale	Very fast (seconds to minutes per ligand); suitable for screening millions of compounds	Slower (ns-µs simulations take hours-days; µs-ms events require HPC/GPUs)
Protein flexibility	Mostly rigid; limited induced-fit handling; ensemble docking partly addresses flexibility	Fully flexible; captures loop motions (activation loop, P-loop, DFG-flip) and induced fit
Solvent treatment	Often implicit or ignored, explicit water is rarely included	Explicit solvent and ions modeled; captures water-mediated hydrogen bonds and dynamic networks
Binding affinity prediction	Approximate; relies on empirical scoring functions; prone to false positives/negatives	More accurate (MM-PBSA, MM-GBSA, FEP); force-field dependent but closer to experimental data
Allosteric site detection	Poor; relies on known crystal structures; limited ability to identify cryptic pockets	Strong, long simulations and enhanced sampling reveal transient/cryptic allosteric sites
Resistance mutation analysis	Limited; requires docking into mutant static structures; cannot capture dynamic effects	Strong; reveals impact of mutations on dynamics, binding, and conformational landscapes
Computational cost	Low; runs on modest hardware; scalable to millions of ligands	High; requires GPUs or HPC for long timescales and enhanced sampling
Best use cases	High-throughput virtual screening; initial pose generation; preliminary selectivity profiling	Refinement and validation of docking poses; binding free-energy ranking; mechanistic insights; exploration of conformational heterogeneity

Docking provides rapid, large-scale screening, while MD, refines binding interactions under dynamic and physiologically realistic conditions.

## 8 Emerging directions in STK drug discovery

Molecular docking and MD simulations have already transformed the landscape of kinase inhibitor discovery, but several of their benefits in the STKs drug discovery pipeline remain to be unraveled. Emerging technologies now offer promising integration opportunities that can address many existing limitations as experimental techniques become increasingly sophisticated in producing comprehensive structural data, and computational methods continue to become more powerful. The next few subsections discuss some of the more important future directions that will likely impact the application of drug discovery focused on STK.

### 8.1 Machine learning and AI integration

The integration of AI in structure-based drug discovery is set to revolutionize inhibitor development (Nair and Weiskirchen, 2024). Over the last few years, ML algorithms have been used to enhance the scoring functions to obtain better estimates of binding affinities compared to conventional empirical or force-field-based approaches (Barradas-Bautista et al., 2023). Deep learning models, including graph neural networks, can directly identify chemical-biological interaction patterns in larger biological datasets, thereby providing better candidates for estimating kinase-ligand binding (Zhou and DiMaio, 2025). From DiffDock to generative chemistry models, recent innovations demonstrate that AI can accurately predict binding poses, as well as the genetic and bio-predictive engineering of new scaffolds optimized for selectivity and potency (DL et al., 2024). In contrast, scoring driven by ML can aid in discriminating isoforms, even when their structures are highly conserved for kinases, thus enhancing kinase selectivity. Moreover, the combination of AlphaFold2 and related structure-prediction tools makes accurate three-dimensional models available for even those kinases whose experimental structures are not yet available, greatly increasing the chemical space that can be explored *in silico*.

### 8.2 Enhanced sampling and conformational exploration

The conformational plasticity of kinases remains a significant challenge in their study. While conventional MD simulations provide valuable insights, they may lack the sampling necessary to fully capture rare but functionally important events that are key to function (e.g., DFG-flips, activation loop unfolding, transient opening of cryptic allosteric pockets). To overcome such limitations, researchers are applying enhanced sampling methods, such as accelerated MD, metadynamics, replica-exchange MD, and Gaussian accelerated MD methods, in an ever-increasing fashion (Wang et al., 2021). Such mapping of the entire conformational landscape of these important signalling proteins by these techniques can expose cryptic binding sites as well as resistance-associated conformations and reveal kinetic information on inhibitor binding. Future work that combines enhanced sampling with Markov state models could potentially expand on this point by providing more clarity on how specific ligands stabilize or destabilize certain states for each kinase, and could offer further insight into the overall mechanism of drug action.

### 8.3 Hybrid quantum mechanics/molecular mechanics (QM/MM) approaches

While molecular mechanics is powerful, classical force fields may lack sufficient accuracy for phenomena such as covalent inhibitor binding, transition-state stabilization, and metal ion coordination (Lodola and De Vivo, 2012). Hybrid quantum mechanics/molecular mechanics (QM/MM) methods fill this void by allowing for quantum-level treatment of the active site of the protein while effectively representing the remainder of the protein at the level of a classical force field (Kulkarni et al., 2022). Although QM/MM provides unique insight into the binding energetics and initiation of catalysis across many systems, this approach is especially well-suited for STKs, where the conserved lysine or the DFG aspartate plays key roles in phosphotransfer and inhibitor recognition. As many quantum algorithms are being developed and computing hardware is improving at a rapid speed, QM/MM will be gradually adopted not just for niche applications, but as part of the routine steps in kinase drug discovery pipelines.

### 8.4 Heterobifunctional degraders (PROTACs) and targeted protein degradation

An important emerging modality beyond classic small-molecule inhibitors is targeted protein degradation using heterobifunctional molecules (PROTACs) (Yu et al., 2021). PROTACs recruit an E3 ligase to the kinase, forming a ternary complex that triggers ubiquitination and degradation; this approach can overcome limitations such as resistance caused by active-site mutations and can target non-enzymatic functions (Guardigni et al., 2023). Computationally, PROTAC design involves sampling and evaluating ternary complexes (kinase-PROTAC-E3) to predict the cooperativity/stability of the ternary assembly (Jiang et al., 2023). Docking and MD have been adapted to this challenge via stepwise docking, flexible linker sampling, and MD-based stability and MM-GBSA estimates for ternary complex energetics. Recent studies have demonstrated that MD-refined ternary modeling, when combined with experimental assays, can effectively prioritize PROTACs with favorable degradation profiles (Kossakowski et al., 2025).

### 8.5 Cloud computing and high-throughput simulations

The movement towards more accessible “cloud-based” or “distributed computing” environments is starting to democratize access to large-scale simulations (Banegas-Luna et al., 2019). Thanks to cloud-native workflows, researchers can carry out high-throughput docking of millions of compounds, followed by MD-based refinement, all without needing local infrastructure (Ghanakota et al., 2020). This method is beneficial for kinome-wide selectivity profiling, which requires evaluating several kinases simultaneously. Cloud computing has the potential to enable smaller laboratories to participate in large-scale collaborative efforts for STK-targeted discovery, allowing for a more comprehensive search of chemical space and faster lead identification. In the future, these types of high-throughput, cloud-enabled approaches are expected to become increasingly common in computational drug discovery as costs continue to decrease and infrastructure matures.

### 8.6 Integration with structural biology

The future of computational kinase drug discovery is a tighter coupling with structural biology. Recent developments in cryo-electron microscopy (cryo-EM), nuclear magnetic resonance (NMR), and structural proteomics are enabling the unprecedented resolution of kinase conformations in near-native environments (Son et al., 2024). These advances are often supplemented, and in some cases made even possible, by progress in computational methods. MD simulations are also capable of refining cryo-EM structures, testing the stability of conformations observed in experiments, and exploring transitions not observable via experiment. Docking can suggest binding hypotheses, which can be confirmed with mutagenesis experiments or crystallographic validation. This cycle of computation and experiment will be crucial for STKs to identify cryptic allosteric sites, gain insights into conformational heterogeneity, and validate the structural predictions computationally.

### 8.7 Overall outlook

**FIGURE 4:**
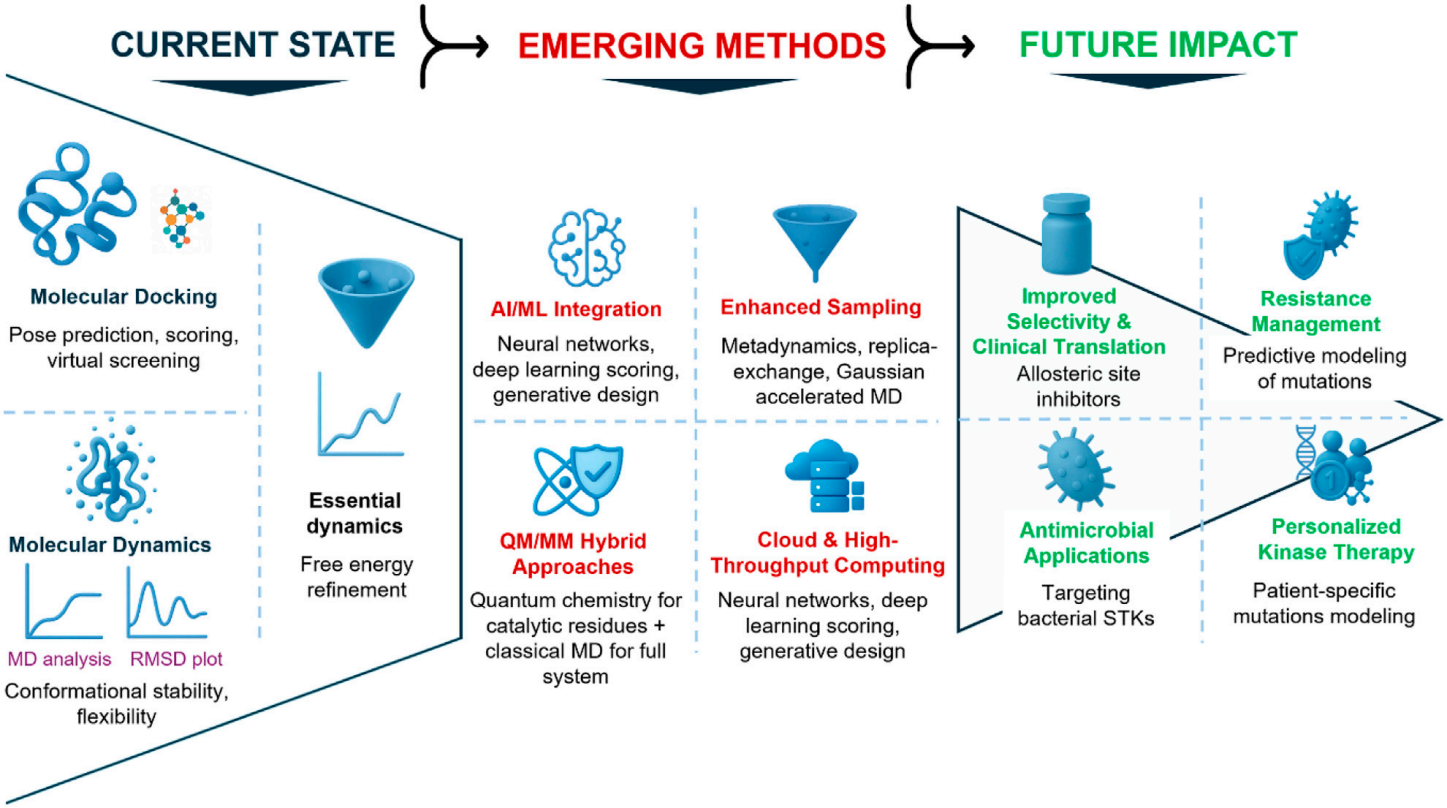
Future perspectives in integrating docking and molecular dynamics for serine/threonine kinase (STK) drug discovery. Illustration of the evolving landscape of computational pipelines. Current strategies (bottom) rely on docking and MD to generate binding hypotheses and refine inhibitor poses. Emerging innovations (middle) include artificial intelligence/machine learning for improved scoring and generative chemistry, enhanced sampling techniques to capture conformational heterogeneity, hybrid QM/MM methods for catalytic modeling, and cloud-based platforms enabling high-throughput simulations. These developments are expected to have future impacts (top), including improved selectivity through allosteric targeting, prediction and management of resistance mutations, antimicrobial applications against bacterial STKs, and personalized kinase therapies. Collectively, these advances will shape the next-generation of kinase inhibitor discovery.

All in all, these emerging strategies will significantly enhance the predictive and translational value of computational drug discovery for STKs. AI will enable more precise scoring and new scaffold generation. Enhanced sampling will expose buried conformations, QM/MM will allow quantum-accuracy at the active site, and cloud computing will scale discovery efforts to the kinome (Kulkarni et al., 2022). Integration with structural biology will test their predictions in physiologically relevant contexts. Together, these innovations represent the next-generation of docking and MD pipelines, which will not only be used to complement new experimental discovery but actually drive the discovery of selective, potent, and sustainable therapeutics against STKs. Figure 4 shows emerging strategies and their predicted translational implications for drug discovery in STKs.

## 9 Conclusion and future prospects

STKs continue to represent the most attractive yet potentially the most challenging targets for drug discovery and development. Altered regulation of these STKs contributes to diseases, including cancer, neurodegeneration, and metabolic disorders in humans, as well as antimicrobial resistance in the microbial world, making them therapeutically relevant in diverse systems in both humans and microbes. Hence, it is essential to identify and validate new classes of small-molecule inhibitors/modulators against new targets using *in silico* and *in vitro* methodologies. In this review, we highlight how molecular docking and MD simulations serve as essential platforms to address these challenges through virtual screening, binding mode prediction, dynamic validation of inhibitor interactions, and exploration of conformational plasticity. These computational approaches hold considerable promise but remain significantly limited by target selectivity, scoring inaccuracies, computational costs, and the difficulty in predicting resistance mutations. However, recent developments, including ML-enhanced scoring functions, accelerated sampling approaches, hybrid quantum-classical computations, cloud computing pipelines, and integrative methods with structural biology, are rapidly expanding their predictive power and accessibility. Moving forward, we envision that an integrated docking–MD–AI framework can be employed in a single discovery pipeline to develop selective, potent, and stable STK inhibitors. These approaches will not only provide quicker ways of drug discovery for human diseases by bridging computational predictions with experimental confirmation but also help in identifying new pathways using bacterial STKs for developing antimicrobial therapies. In this manner, computational approaches are poised to have a significant impact on the future of kinase-based therapies. However, combining *in silico* with *in vitro* validation is essential to avoid over-reliance on computational predictions. Overall, the synergy between computational and experimental techniques will continue to accelerate the rational design of STK-targeted therapeutics.
